# Epigenomic Mechanisms of Early Adversity and HPA Dysfunction: Considerations for PTSD Research

**DOI:** 10.3389/fpsyt.2013.00110

**Published:** 2013-09-26

**Authors:** Patrick O. McGowan

**Affiliations:** ^1^Centre for Environmental Epigenetics and Development, Toronto, ON, Canada; ^2^Department of Biological Sciences, University of Toronto, Scarborough, ON, Canada; ^3^Cell and Systems Biology, University of Toronto, Toronto, ON, Canada

**Keywords:** epigenetics, DNA methylation, early adversity, childhood abuse, brain development, hypothalamic-pituitary-adrenal axis, stress response, glucocorticoid receptor

## Abstract

Childhood adversity can have life-long consequences for the response to stressful events later in life. Abuse or severe neglect are well-known risk factors for post-traumatic stress disorder (PTSD), at least in part via changes in neural systems mediating the endocrine response to stress. Determining the biological signatures of risk for stress-related mental disorders such as PTSD is important for identifying homogenous subgroups and improving treatment options. This review will focus on epigenetic regulation in early life by adversity and parental care – prime mediators of offspring neurodevelopment – in order to address several questions: (1) what have studies of humans and analogous animal models taught us about molecular mechanisms underlying changes in stress-sensitive physiological systems in response to early life trauma? (2) What are the considerations for studies relating early adversity and PTSD risk, going forward? I will summarize studies in animals and humans that address the epigenetic response to early adversity in the brain and in peripheral tissues. In so doing, I will describe work on the glucocorticoid receptor and other well-characterized genes within the stress response pathway and then turn to genomic studies to illustrate the use of increasingly powerful high-throughput approaches to the study of epigenomic mechanisms.

## Introduction

Childhood adversity can have life-long consequences for the response to stressful events later in life (1). Repeated exposure to trauma alters neurodevelopment (2), enhances the activity of endocrine mechanisms involved in the stress response (3, 4) and increases the risk of multiple forms of psychopathology (5, 6). For example, the risk of suicide is strongly linked to childhood sexual and physical abuse or severe neglect (7–9). Sexual and physical abuse or severe neglect in childhood are also well-known risk factors for adult forms of post-traumatic stress disorder (PTSD), at least in part via changes in neural systems mediating the endocrine response to stress (10). The hypothalamic-pituitary-adrenal (HPA) axis shapes the endocrine response to stress in addition to its role in many other physiological processes, including immune and metabolic function. As such, the HPA axis plays an adaptive role by maintaining allostasis (i.e., stability amid change) in the face of challenging environmental conditions. Part of the explanation for the enhanced impact of adversity in early life is thought to lie in the relatively high degree of plasticity during this period, when environmental factors exert pervasive effects on a number of health trajectories (11, 12). Accumulating evidence indicates that this phenomenon, sometimes called “biological embedding,” involves persistent changes in gene regulation via epigenetic mechanisms (13). The goal of this review is to highlight research on epigenetic mechanisms of early life adversity and parental care – prime mediators of offspring neurodevelopment (11) – that addresses several critical issues for research in this rapidly evolving area. We conclude by providing examples of the ways in which research in this area may provide insights for PTSD researchers on the epigenetic impacts of early adversity and highlight challenges for the field going forward.

## Epigenetic Mechanisms: Stability and Change

A first critical issue in understanding the relative risk conferred by early life adversity concerns the molecular mechanisms mediating altered HPA function as well as other pathways underlying vulnerability that respond in a manner that is both contingent upon the adversity and stable in the face of similar perturbations in later life. Epigenetic mechanisms include DNA methylation, histone modifications, and non-coding RNA. The methylation of cytosine in cytosine-guanine dinucleotides (CpGs) in the DNA itself (i.e., 5meC) is the best understood epigenetic mark and the focus of the majority of current investigations. However other modifications to DNA, including hydroxymethylation (5-hmC) and other recently identified DNA modifications, are attracting increasing interest as potential gene regulatory mechanisms (14). It should be noted that the conventional methods used for mapping 5-mC, such as bisulfite sequencing and methylation-sensitive restriction enzyme-based approaches, do not differentiate it from 5-hmC. As such, although I use the term “DNA methylation” in this review to be consistent with the majority of primary publications to date, the term “DNA modification” is a more accurate descriptor. Variations in these modifications occur as a result of genetic, stochastic, and environmental factors, all of which drive the epigenetic regulation of gene expression. There is some debate as to the primacy of stochastic and environmental factors in epigenetic variation (15). It is clear that proper epigenetic regulation is essential for normal development and cell division, conferring cell-type identity in a stable manner that appears to a large degree unresponsive to early life adversity. There also is now compelling evidence of epigenetic regulation by environmental factors. Epigenetic regulation thus provides a potential mechanism for understanding well-defined environmental effects on phenotypes.

Elucidating which regions of the genome are labile in response to early life adversity, how rapidly changes can occur, and the ontological time-course of epigenetic changes remains a matter of active investigation. As I discuss below, these epigenetic responses likely depend on the genomic loci under consideration. Humans are exposed to a variety of stressors throughout life, however early life stress appears to exert an profound effects on HPA function that is pervasive throughout life in part by altering epigenetic mechanisms in a stable manner. I will illustrate this point by discussing several studies in rodents that have provided foundational knowledge applicable to investigations in humans.

## Animal Models of Epigenetic Mechanisms in Early Life Shaping the Response to Stress in Adulthood

Animal models of maternal care and perinatal stress have helped to provide a mechanistic understanding of the impacts of early life adversity, allowing for control of genetic variation and a temporal dynamics of environmental exposures. Classic examples are experiments pioneered by Levine in the late 1960s and Meaney beginning in the late 1980s that indicated that laboratory rodents exposed to different levels of maternal care show behavioral alterations in fearfulness in response to novel environments and endocrine-mediated stress responses (16). These studies have documented sustained alterations in the expression genes regulating HPA function, such as the Glucocorticoid Receptor (GR), in brain areas mediating anxiety behavior and HPA circuitry, such as the prefrontal cortex, hippocampus, and hypothalamus. As adults, the offspring of rat mothers providing relatively high or relatively low levels of maternal care display life-long alterations in DNA methylation and Histone 3 lysine 9 (H3K9) acetylation of the untranslated 1_7_ splice variant of the GR promoter in the hippocampus and of the promoter of the GAD67 gene in the prefrontal cortex (17, 18). Other groups have provided evidence that additional genes in neural pathways mediating the stress response are epigenetically regulated in association with early life stress, including arginine vasopressin in the hypothalamus (19), and BDNF in the prefrontal cortex and hippocampus (20). Interestingly, apparently stable changes in GR promoter methylation emerge within the first week of life as a function of naturally occurring variations in maternal care. However, a recent study found evidence of sex-specific DNA methylation changes in BDNF and reelin in the medial prefrontal cortex of offspring subjected to an adverse maternal environment that emerge in the transition between adolescence and adulthood (21). These data indicate a complex temporal relationship between environmental adversity and epigenetic variation in the medial prefrontal cortex, dependent upon unknown mediating factors. The data suggest that the temporal dynamics of the epigenetic response to early adversity may, at least to some extent, be loci- and tissue-specific.

## Human Studies of Epigenetic Mechanisms in Early Life Shaping the Response to Stress in Adulthood

In light of findings in animal models, GR is an obvious candidate gene of interest in exploring the relationship between epigenetic regulation as a function of early life adversity and mental health outcomes in humans. Perhaps less clear is the choice of appropriate cohorts and cell types in humans to test these relationships. As mentioned, epigenetic mechanisms play an important role in conferring cell-type identity during development and cell division. As a result, it is perhaps reasonable to assume that the impact of environmental factors on epigenetic marks is likely to be to some extent cell-type specific, limiting analysis to appropriate tissues of interest. We used hippocampal samples from suicide completers with and without a history of childhood abuse, and examined DNA methylation of the GR1_F_ promoter, a region highly syntenic with the rat GR1_7_ splice variant. We found higher levels of DNA methylation of the GR promoter region among suicide victims with a history of abuse or severe neglect in childhood, but not among suicide victims who were not abused in childhood or among a control group who had died of causes unrelated to suicide (22). This hypermethylation was associated with increased transcript abundance of both GR1_F_ splice variant and total abundance of GR transcript, and *in vitro* analysis indicated that regions hypermethyated in abused suicide victims inhibited the binding of the EGR1 transcription factor (also known as NGFI-A, Zif268, Krox24, and ZENK) to select nucleotides within the promoter. Another recent study has replicated the finding of enhanced DNA methylation at this splice variant and gone on to identify altered DNA methylation in additional splice variants of the GR promoter and show that this response to early adversity is brain region specific, not occurring in the anterior cingulate (23).

## Studying Epigenetic Mechanisms of HPA Regulation by Early Adversity in Peripheral Tissues in Humans

A second important consideration for studies of the epigenetic response to early life adversity in living humans is its impacts on peripheral tissues, essential for efforts to sample potential changes over time and after interventions in humans. Lymphocytes are well-known targets of glucocorticoids, and immune profiles are known to be sensitive to alterations in GR abundance (24). One study found that childhood adversity (as measured by parental loss, childhood maltreatment, and parental care) was associated with increased DNA methylation of several sites within the GR1F promoter region in lymphocytes in adulthood (25). These results and other analogous data are important because they indicate that epigenetic alterations as a result of childhood adversity persist in peripheral tissues and are detectable in mixed lymphocyte cell populations. A recent investigation in whole blood of FKBP5, a negative regulator of GR, links PTSD to both genetic variation and early adversity (26). The authors of this study had previously characterized several genetic polymorphisms associated with PTSD risk. In the recent study, they found evidence of DNA demethylation in an intronic region only in individuals subjected to abuse in childhood and only in those carrying the “risk” allele of the gene, with experiments in cultured cells indicating an effect shown to occur before and persist after differentiation in cultured hippocampal cells. In light of previous animal work showing that glucocorticoid exposure can drive DNA demethylation in mouse hippocampal dentate gyrus, indicating neural target tissues and *in vivo* conditions where glucocorticoid activity may modulate other HPA-responsive genes (27). These data investigating candidate genes demonstrate the capacity of the epigenetic machinery to respond to the psychosocial environment in early life in a manner that confers stable changes in stress pathways in lymphocytes – cells that evidently go through numerous cycles of cell division throughout life.

## Epigenomic Regulation by Early Life Adversity in Gene Regulatory Elements and Beyond

A third consideration addressed by these studies is the need to identify genomic loci that are epigenetically labile in response to early life adversity. Studies to date have predominantly focused on epigenetic changes in gene regulatory elements (e.g., promoters) and defined candidate genomic loci. A study using a microarray approach combined with methylated DNA immunoprecipitation to interrogate promoter regions in all known protein-coding genes found that evidence of hypo- and hypermethylation among hundreds of genes in hippocampi from suicide completers with a history of early life abuse compared to non-abused controls (28). This study identified novel candidate genes (e.g., ALS2; involved in small GTPase regulation) and enriched candidate pathways (e.g., neuroplasticity) that may be epigenetically regulated in response to early life abuse and suicide. Another study of whole blood using the Illumina 450 K array, which examines the methylation status at single-nucleotide resolution in ∼480,000 CpG sites, covering most known genes and regulatory elements, found evidence of predominantly hypermethylated DNA within exons and 3′ UTRs of differentially expressed genes in PTSD patients with a history of early abuse, with epigenetic differences showing general agreement with levels of transcription (29). This study indicated that changes in DNA methylation among PTSD patients were enhanced in a with a positive history of childhood abuse, suggesting a potentially distinct epigenetic profile in this subgroup.

We documented changes in DNA methylation, H3K9 acteylation and gene expression across a 7 Mb region flanking the GR gene hippocampus using a tiling microarray approach in rats (30). Differences in the amount of maternal care received during the first week of life were associated with epigenetic differences over large genomic regions (∼100 kB) in hippocampi of adult animals. Differences in transcription occurred in the context of hyperacetylation and hypomethylation of promoters and hypermethylation of exons. Interestingly, hypermethylation within exons was the largest detect difference in DNA methylation as a response to higher levels of maternal care. Using this methodology, we identified a novel linkage between altered epigenetic status of a large protocadherin (PCDH) gene cluster of cell-adhesion molecules and maternal care. Previous studies have indicated that PCDH gene clusters regulate neuronal morphology and synaptic plasticity (31). It remains to be determined whether epigenetic alterations in these genes are linked to differences in neuroplasticity observed as a function of differences in maternal care (32). Nevertheless, as technologies for generating genome-wide epigenetic profiles become economically accessible to a wider array of researchers and bioinformatics tools for genomic analysis become more standardized, these approaches will likely provide powerful methods for hypothesis generation by consolidating multiple levels of biological information.

In a follow-up to this study, we analyzed the GR locus in hippocampi of adult suicide victims who were abused early in life compared to non-abused controls (33). Abused suicide victims showed broad statistical dependencies in DNA methylation differences in a manner akin to what was observed in the rat study described above (30). As in the previous study, the clustered PCDH gene cluster showed the largest alterations in DNA methylation within the locus examined. In humans, alterations in PCDH genes impair intellectual function, and mutations in PCDH genes are linked to autism (34). PCDH genes show evidence of distinct DNA methylation in whole blood from individuals with a childhood history of low socio-economic (35). The function of these epigenetic differences in PCDH remains unknown, however the data suggest that these genes are epigenetically labile in response to the early life social environment in both rodents and humans (33). Taken together, the data suggest that animal model of parental care may have broad applicability for understanding the consequences of epigenetic modification of PCDH gene pathways in humans.

An important caveat of these studies is that they often report data from mixed cell populations, potentially masking epigenetic differences in select cell types or skewing group differences due to cell admixture. Fluorescence-associated cell sorting followed by cell-type-specific epigenomic analysis is a potential solution. However, the relevant cell types are not often known, and cell types that are routinely extracted (e.g., CD4^+^ T-cells) can often be divided into functional classes that are dissociable by additional rounds of selection, making it difficult to know whether one has attained the necessary level of specificity. An additional method to address this problem is informatic. Data gathered by the Encyclopedia of DNA Elements (ENCODE) project and other large-scale genomics initiatives are providing multidimensional representations of epigenetic and functional genomic signatures from a large number of cell types (36). These data will serve as important information on regions that identify cell types that can be used to bioinformatically deconvelute the constituents of cell admixture in mixed tissue populations [e.g., peripheral blood; (37)]. The data will also provide a valuable method to identify epigenetically invariant genomic regions that can serve to reduce genomic complexity in genome-wide analysis of epigenetic signaling, and transcriptional “silent” regions in specified cell types unlikely to be responsive to environmental perturbations. These data, together with an accumulating array of published epigenomic analysis, should help move research on the impacts of early life adversity beyond candidate gene to “candidate pathway” and “candidate network” levels of analysis, which are finding utility in other areas of complex disease research [e.g., (38)].

## Prospective for PTSD Research

Early life trauma shapes resiliency to stress in later life and is a risk factor for the development of PTSD, itself characterized by a “transformational” change in the neurophysiological response to stress that occurs in some but not all individuals exposed to trauma (39). Inter-individual differences in PTSD susceptibility are modulated at least to some extent by early life adversity inasmuch as both are associated with HPA axis alterations – at least in a subset of PTSD patients. Both early life trauma or severe neglect and PTSD are generally associated with lower basal circulating cortisol levels and an attenuated response to acute stress challenge (10). These results have been proposed to explain a paradox of PTSD: namely that HPA dysfunction observed in PTSD appears distinct from that observed in chronic stress or major depression, conditions associated with *elevated* levels of cortisol. Because PTSD and major depression co-occur ∼50% of the time, the results indicate a distinct profile of PTSD in patients with a past history of trauma or early life abuse (10). Likewise, not all who experience trauma develop PTSD. A few studies have identified epigenetic variation associated with PTSD [e.g., (40)], and patients with a history of early life adversity may show distinct epigenomic profiles (29). These contrasts have made it challenging to identify epigenetic mechanisms linking early adversity to PTSD risk, calling for a variety of approaches in appropriate animal models and human studies. The molecular and epigenetic mechanisms associated with PTSD with and without a history of early life adversity are beyond the scope of the present manuscript, however this topic has been the focus of a number of excellent reviews [e.g., (10, 41–43)] – including in this volume (44).

Questions that need to be addressed for a more complete understanding of the role of epigenetic mechanisms in conferring risk of PTSD via early life adversity, include: when, precisely, during development, do epigenetic changes related to early adversity emerge? In what contexts, genomic regions/pathways, and in cell types? These principles remain poorly understood. However, some interesting parallels have been identified between regions of the genome that are epigenetically responsive to psychosocial factors (e.g., maternal care) in rodents, and syntenic regions of the human genome that are epigenetically labile in conditions of early adversity [e.g., childhood abuse; (33)]. Studies in animal models have suggested that early life stress impairs neuroplasticity in brain regions such as the hippocampus and has a lasting impact on endocrine systems underlying the response to psychosocial stressors (45, 46).

Many animals, including rodents and humans, appear to have evolved to respond both to immediate threats to life and limb and to psychosocial stress associated with predation risk, including via the transfer of information about environmental conditions to the offspring via maternal factors. For example, a number of studies in wildlife ecology and comparative endocrinology over the past 20 years have indicated that the influence of predators on stress in free-living animals is long-lasting, resembling stress effects in laboratory animal models of PTSD (47). Response mechanisms mediating the adaptive processes responsible for this transmission implicate the HPA axis and pathways involved in neuroplasticity (48, 49). Epigenetic research in this area is in its infancy, but offers an important avenue to study the extent to which developmentally regulated epigenetic mechanisms and environmental stressors interact in the context in which they have evolved.

Elucidating the biological mechanisms underlying effects of early social experiences on later mental health is challenging in humans for reasons that include technical/analytic complexity and limited access to relevant biological material. New methods that offer the ability to examine DNA methylation at single-nucleotide resolution genome-wide are advancing rapidly and, in tandem, a vast array of analytical tools and statistical methods are now available to normalize known technical biases, visualize epigenetic modifications, and identify differences among subjects (50). Genome-wide changes with early adversity appear to occur in association with pathway or network-specific alterations of the epigenomic landscape. Thus, the selection of epigenetic modification(s) for study and identification of the impacted pathways, which rely on computationally predicted and biologically validated relationships, remain a challenge for future studies. The use of whole-genome screens to identify stable combinations of epigenetic modifications that distinguish cell- or tissue-specific functional effects may be useful in tissue-specific gene targeting of therapeutics while minimizing off-target effects (51). It may not be clear, however, which cell types are relevant to the question under study. Nevertheless, there is some indication that even buccal epithelial cells may index the response to early life adversity, though not via epigenetic changes in GR *per se* (52). Buccal cells share embryonic stem cell origin with neurons, and therefore may provide a valuable means of identifying the epigenetic signature of early life adversity in young children, where blood sampling is problematic. In addition, because changes in epigenetic patterns are often only measured at one time-point, the involvement of later life experiences in conferring epigenetic changes are difficult or impossible to rule out. Prospective research validating the use of peripheral markers of early life impacts (which can also be done in animal models) will offer critical insights into the dynamic nature of epigenetic regulation and its role as a mechanism for programing gene function in response to early life trauma.

## Conflict of Interest Statement

The author declares that the research was conducted in the absence of any commercial or financial relationships that could be construed as a potential conflict of interest.
